# Thermodynamic Properties, Viscosity, and Structure of CaO–SiO_2_–MgO–Al_2_O_3_–TiO_2_–Based Slag

**DOI:** 10.3390/ma14010124

**Published:** 2020-12-30

**Authors:** Jinle Fang, Zhuogang Pang, Xiangdong Xing, Runsheng Xu

**Affiliations:** 1 School of Metallurgical Engineering, Xi’an University of Architecture and Technology, Xi’an 710055, China; wangqi@xauat.edu.cn (J.F.); hjpzg@xauat.edu.cn (Z.P.); 2 Shannxi Steel Group Hanzhong Iron and Steel Co., Ltd., Hanzhong 724200, China; 3 School of Metallurgical and Ecological Engineering, University of Science and Technology Beijing, Beijing 100083, China; xurunsheng@ustb.edu.cn

**Keywords:** heat capacity, enthalpy change, viscosity, activation energy, structure

## Abstract

The effect of TiO_2_ and the MgO/Al_2_O_3_ ratio on the viscosity, heat capacity, and enthalpy change of CaO–SiO_2_–Al_2_O_3_–MgO–TiO_2_ slag at constant heat input was studied. The variation of slag structure was analyzed by the calculation of activation energy and FTIR spectrum measurements. The results showed that the heat capacity and enthalpy change of the slag decreased with the increase of TiO_2_ content. Under constant heat supply, the fluctuations in slag temperature were relatively apparent, and the temperature of slag increased as the TiO_2_ content increased. The viscosity of slag decreased due to the increase in slag temperature. Increasing the MgO/Al_2_O_3_ ratio could decrease the temperature and viscosity of slag. The effect of increasing the MgO/Al_2_O_3_ ratio on the viscosity was more pronounced than the decreasing temperature caused by increasing the MgO/Al_2_O_3_ ratio. The apparent activation energy decreased with increasing TiO_2_ content and MgO/Al_2_O_3_ ratio. The Ti–O bonds formed with TiO_2_ addition, and the Ti–O bonds were weaker than Si–O bonds, which resulted in the decrease in heat capacity and viscosity of slag.

## 1. Introduction

The viscosity of slag is a vital factor in blast furnace (BF) smelting and has a pivotal role in gas permeability, BF smooth operation, and reduction of iron oxide [1,2,3,4]. The viscosity of slag is affected by its composition. The reserves of vanadium-titanium magnetite (VTM) ores are relatively abundant in China and Australia, and the VTM ores usually contain significant TiO_2_ [5]. These ores have been used in BF smelting, and as a result the BF slag contains an amount of TiO_2_. In addition, many steel enterprises adjust the MgO content to improve the fluidity of slag since the high aluminum content of slag increases its viscosity. Both aspects have a great impact on the viscosity and thermal stability of slag. Therefore, it is of great significance to study the effect of TiO_2_ and the MgO/Al_2_O_3_ ratio on the viscosity of BF slag.

Research related to the effect of the slag component on the viscosity have been broadly conducted. Sohn et al. [6] studied the effect of TiO_2_ from 0 to 10 wt.% on the viscosity of calcium silicate molten slag containing 17 wt.% Al_2_O_3_, where TiO_2_ depolymerized the silicate network structure and lowered the viscosity. Liao et al. [7], Zhang et al. [8], and Yan et al. [9] also investigated the viscosities of BF slag with varying TiO_2_ content, and the results showed TiO_2_ reduced the apparent viscosity of molten slags. Zhang et al. [10] revealed the influence of the MgO/Al_2_O_3_ ratio on the viscosity of high-Al BF slag and recommended the proper ratio in a range of 0.6–0.7. Although the effect of TiO_2_ and the MgO/Al_2_O_3_ ratio on the viscosity of BF slags at a constant temperature has been investigated, limited research has been conducted at a fixed heat quantity supply. During the BF operation, the fuel ratio and furnace conditions are basically steady; that is, the heat quantity supply in a blast furnace is steady. However, the composition of slag affects its heat capacity, which leads to the different temperature and viscous behavior of slag. Hence, it is of great importance to investigate the relation between the thermodynamic properties of slag with different compositions and its viscosity.

In this study, the viscosity of BF slag with varying TiO_2_ content and MgO/Al_2_O_3_ ratios was measured. According to the thermodynamic data, the heat capacity and enthalpy change of slag were calculated. In addition, the variation of the slag structure with different TiO_2_ contents was analyzed using Fourier Transform Infrared (FTIR) spectroscopy.

## 2. Materials and Methods

### 2.1. Thermodynamic Calculations

The compositions of experimental slags at fixed basicity (C/S = 1.10) are listed in Table 1, and the mass of each sample in the process of calculations was set as 100 g. Factsage thermodynamic software (version 7.1, CRCT & GTT, Montreal, QC, Canada) was used to calculate the heat capacity and enthalpy change of slag, and then the results were corrected according to Equations (1)–(9) to eliminate the unavoidable influence of chemical reaction, crystal transition, and phase change heat. In addition, the liquid temperature of each group of the slags was also calculated using Factsage to ensure that the heat capacity and enthalpy change were calculated in the complete liquid phase region. Factsage is one of the largest computing systems in the chemical thermodynamics field and has been widely used to calculate the thermodynamic data of molten slag [11,12,13].

In the process of calculations, the initial conditions were set as 298 K and 101,325 Pa. The heat capacity and enthalpy change of slag at 1723, 1773, 1823, and 1873 K were calculated. Theoretically, when the pressure of a system is constant and the non-volume work is not conducted, the enthalpy change of slag is approximately equal to the heat absorbed by slag, which is known from the first law of thermodynamics. The average enthalpy changes of slag with varying TiO_2_ content and MgO/Al_2_O_3_ ratios at 1773 K and 1823 K were calculated to use as the given heat quantity supply, and then the temperature of slag at the corresponding heat input was calculated.

C_pCaO_ = 1.048 − 2.046 × 10^4^ T^−2^ − 2.388 T^−1/2^ + 1.836 × 10^6^ T^−3^ (298 K to 2845 K) (1)

C_pSiO_2__ = 1.332 − 5.903 × 10^4^ T^−2^ − 3.999 T^−1/2^ + 8.181 × 106 T^−3^ (298 K to 1996 K) (2)

C_pMgO_ = 1.516 − 1.541 × 10^4^ T^−2^ − 7.349 T^−1/2^ + 1.45 × 10^4^ T^−3^ (298 K to 3098 K) (3)

C_pAl_2_O_3__ = −0.1541 − 2.973 × 10^−4^T − 4.899 × 10^4^ T^−2^ − 1.099 × 10^3^ T^−1^ + 69.33T^−1/2^ (298 K to 1200 K) (4)

C_pAl_2_O_3__ = −7.724 + 6.461 × 10^−4^ T + 2.587 × 10^6^ T^−2^ − 1.496 × 10^4^ T^−1^ + 6.56 × 10^2^ T^−1/2^ (1200 K to 2327 K) (5)

C_pTiO_2__ = 0.975 − 4.217 × 10^4^ T^−2^ + 5.045 × 10^6^ T^−3^ (298 K to 2130 K) (6)

(7) $$C_{p}\;=\;\sum m_{i}C_{\mathrm{pi}}$$

(8) $$\Delta H_{i}\;=\;\int_{298}^{T_{\mathrm{tr}}}C_{\mathrm{pi}}\mathrm{dT}\;+\;\Delta_{\mathrm{tr}}\mathrm{Hi}\;+\;\int_{T_{\mathrm{tr}}}^{T_{M}}C_{\mathrm{Pi}\left(s\right)}^{\prime }\mathrm{dT}\;+\;\Delta_{s}^{l}\mathrm{Hi}\;+\;\int_{T_{M}}^{T}C_{\mathrm{Pi}\left(l\right)}^{\prime }\mathrm{dT}$$

(9) $$\Delta H_{T}\;=\;\sum m_{i}\Delta H_{i}$$

where C_pi_ is the specific heat capacities of component i, J/g·K; i is the slag components of CaO, SiO_2_, MgO, Al_2_O_3_, and TiO_2_, J/g·K; C_p_ is the thermal capacity of the slag at a certain temperature J/K; m_i_ is the mass of component i, g; ΔH_i_ is the enthalpy change of component i at the target temperature; $\Delta_{\mathrm{tr}}\mathrm{Hi}$ is the crystal transition enthalpy of component i at the target temperature, J/g; $\Delta_{s}^{l}\mathrm{Hi}$ is the melting enthalpy of component i at the given temperature, J/g; $\Delta H_{T}$ is the enthalpy change of slag at a given temperature, kJ; T_tr_ and T_M_ are the crystal transition temperature and melting temperature, respectively, K.

### 2.2. Experimental Materials

**Figure 1:**
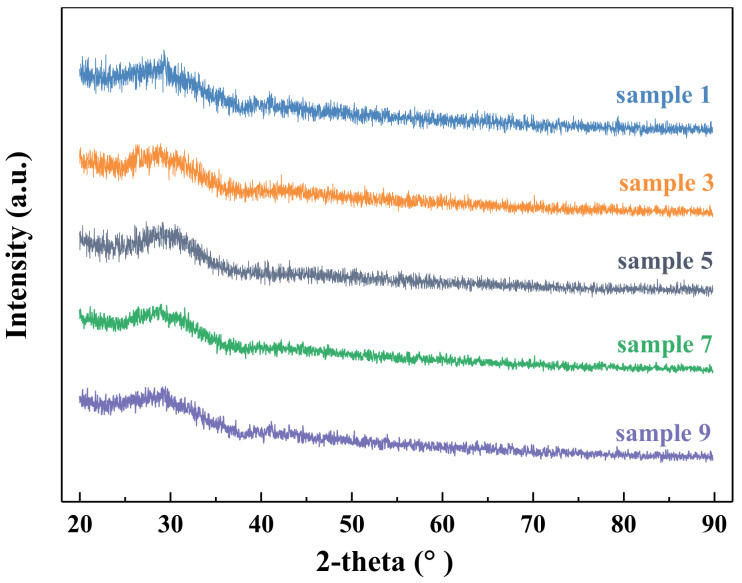
The XRD pattern of CaO–SiO_2_–Al_2_O_3_–CaO–MgO–TiO_2_ slag.

The slags were prepared with the analytical reagents CaO, SiO_2_, MgO, Al_2_O_3_, and TiO_2_ based on the design of slag. All the reagents were heated in an electric resistance furnace at 1273 K for 150 min to remove impurities. The mixture was precisely weighed at 180 g and packed into a Mo crucible. The samples were heated to 1873 K in a muffle furnace with high-purity argon gas (99.99%, 0.5 L/min). After 120 min heat preservation, the slag was rapidly quenched in distilled water, and the cooled slag sample was taken out and dried. All quenched slags were colorless and transparent, which suggested the main valence state of Ti ions is Ti^4+^. The quenched slag was ground and then analyzed by X-ray diffraction (XRD, MAXima-7000, Shimadzu, Kyoto, Japan) that used Cu Kα radiation (λ = 1.5406 Å) with the scan rate of 6°/min in the 2 degree range of 10° to 90°. Figure 1 shows the XRD pattern of the representative slag samples. It could be seen that there were no characteristic peaks, which proved that the quenched slag was amorphous. FTIR spectroscopy (Nicolet 6700; Thermo Fisher Scientific, Waltham, MA, USA) was used to analyze the slag structure. During the measurement, 3.0 mg quenched slag powders and 350 mg KBr were mixed and pressed into a film. The scanning time was set as 32 s. The wavenumber range of 400–4000 cm^−1^ was recorded with a resolution of 4 cm^−1^. 

### 2.3. Viscosity Measurement

**Figure 2:**
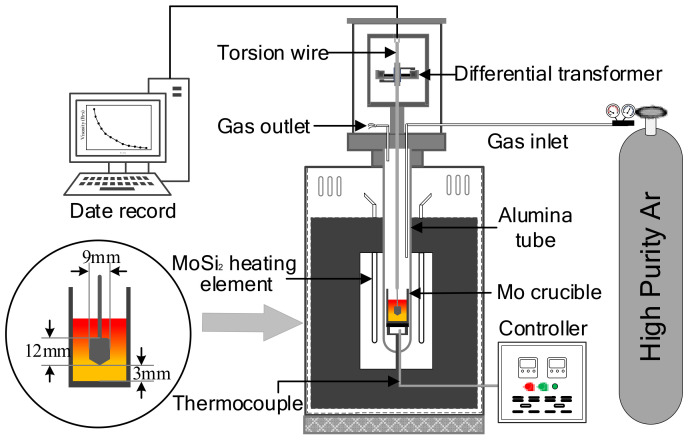
Experimental apparatus for the measurement of slag viscosity.

The viscosity measurements were conducted with the rotating spindle method. The experimental apparatus used to measure the viscosity of slags was shown in Figure 2. Using seven MoSi_2_ heat elements to heat the furnace, a Pt–6%Rh and Pt–30%Rh thermocouple was used to monitor and control the furnace temperature (error ± 2 K). Then, the computer calculated and recorded the viscosity values when the spindle was rotating.

The Mo crucible filled with 110 g of pre-melted slag was put at the constant temperature zone of the furnace. Then, the temperature of the furnace was heated to 1873 K under the protection of argon gas and held for 90 min to ensure the slag was in the fully liquid phase. The spindle was carefully adjusted to the central axis line of the crucible and immersed into the fluid slag. Viscosity measurements were carried out at every 10 K from 1873 K. In order to ensure sufficient thermal equilibration, the furnace was held for 10 min before every measurement. The measurements were ended when the viscosity value of the melt was > 5 Pa·s. The average of three test results was used as the final viscosity value.

## 3. Results and Discussion

### 3.1. Effect of TiO_2_ on Viscosity, Heat Capacity and Enthalpy Change of Slag 

**Figure 3:**
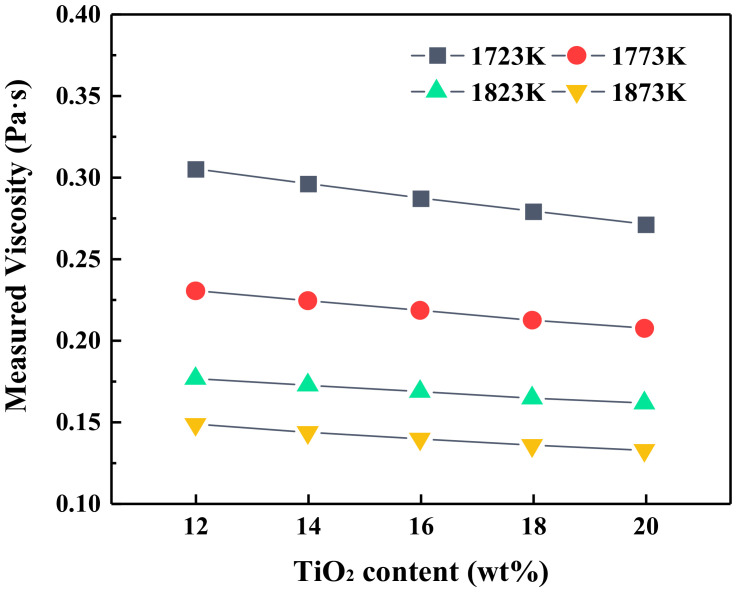
The viscosity of blast furnace (BF) slag with different TiO_2_ contents.

Figure 3 graphically represents the viscosity of slag with different TiO_2_ contents at constant basicity of 1.10. It could be seen from Figure 3 that the slag viscosity decreased with the increment in temperature and TiO_2_ content. TiO_2_ had little effect on the decrease in viscosity at experimental temperatures, which is supposed to be due to the intricate network structure that has been depolymerized into simple units under high temperature [14,15]. The variation trends of viscosity are consistent with many previous works on different slag systems. Zheng et al. [16] investigated the effect of TiO_2_ content (0–30 wt.%) on the viscosity of CaO–SiO_2_–TiO_2_ slag at fixed basicity (C/S = 1.2) and concluded that the TiO_2_ additions weakened the strength of the silicate network and then reduced the viscosity. Jiao et al. [17] showed that the viscosity of CaO–MgO–Al_2_O_3_–SiO_2_–TiO_2_–FeO BF primary slags decreased with increasing TiO_2_. It is generally considered that the variation in slag viscosity is closely related to the slag structure. Thus, the decrement in viscosity might be attributed to the existence of numerous silicates or silicate-aluminate structural units in the present melts, and TiO_2_ may break up the 3D networks consisting of Si–O or Al–O, which results in decreasing the degree of polymerization (DOP) of slag.

**Figure 4:**
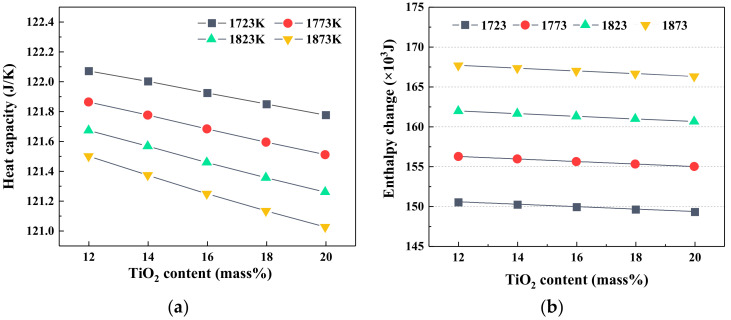
The heat capacity and enthalpy change of slag with varying TiO_2_ contents; (**a**) heat capacity; (**b**) enthalpy change.

The heat capacity and enthalpy change of slag with varying TiO_2_ contents are shown in Figure 4. It could be observed from Figure 4a that the heat capacity of slag presented a linear decreasing trend as the TiO_2_ content increased at the target temperature, and it also decreased as the temperature increased at a constant TiO_2_ content. Meanwhile, when the temperature rose from 1723 K to 1873 K, the decrease rate of heat capacity augmented with increase in TiO_2_ content. Figure 4b shows the enthalpy change of slag as a function of TiO_2_ content at different temperatures. It could be seen that the enthalpy change decreased slightly with the increase in TiO_2_ content and increased dramatically with increasing temperature. The heat capacity and enthalpy change of slag are the important physicochemical properties of slag. The higher the heat capacity, the more heat energy slag needs to absorb when its temperature rises by one degree Celsius, which means that the temperature of slag with a larger heat capacity is difficult to change as the heat quantity supply varies. Therefore, the low TiO_2_ content in slag is beneficial to keep the temperature of slag constant. As mentioned earlier, the heat absorbed by the slag is approximately equal to the enthalpy change value of the slag, so the increase in TiO_2_ content and the decrease in temperature could decrease the heat storage capacity of slag. It was suggested that when the TiO_2_ content increased in the range of 12 to 20 mass%, the heat supply of the BF should maintain stability due to the large fluctuation of slag temperature.

**Figure 5:**
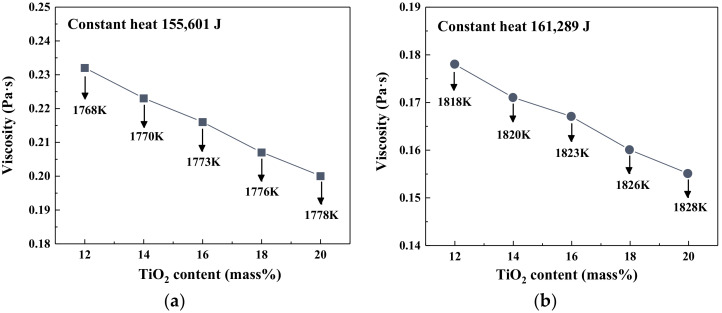
The viscosity of slag with varying TiO_2_ content under constant heat quantity: (**a**) 155,601 J; (**b**) 161,289 J.

The effects of TiO_2_ on the viscosity and temperature of slag at the fixed heat quantity supply are depicted in Figure 5. The value of constant heat input was determined by the average value of the enthalpy change of slag with varying TiO_2_ content at 1773 K and 1823 K. The calculated heat quantity supplies were 155,601 J and 161,289 J, respectively. An increment in the TiO_2_ content led to higher slag temperature and lower slag viscosity. Due to the heat storage capacity of slag decreasing as TiO_2_ content increased, the increase in residual heat raised the slag temperature, which had a significant influence on the network structure of slag [14]. By comparing Figure 3 and Figure 5, it also could be observed that the viscosity under the constant heat quantity supply changed relatively greater than the one at the fixed temperature. Therefore, when the TiO_2_ content in BF slag increases, it is essential to maintain a steady heat quantity input to avoid greater fluctuations in slag temperature and fluidity.

### 3.2. Effect of the MgO/Al_2_O_3_ Ratio on Viscosity, Heat Capacity, and Enthalpy Change of Slag

**Figure 6:**
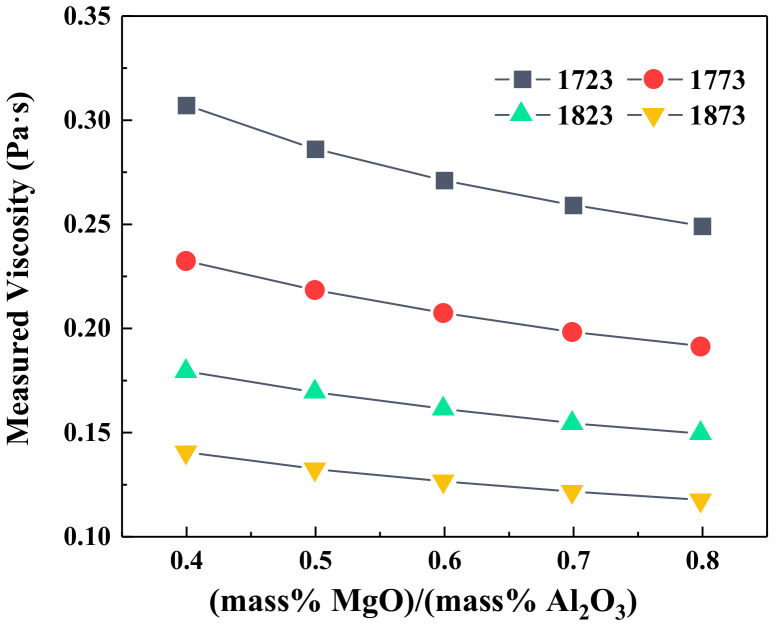
The viscosity of BF slag with different MgO/Al_2_O_3_ ratios.

Figure 6 shows the effect of MgO/Al_2_O_3_ ratios on the viscosity of slag at different temperatures. The increase in MgO/Al_2_O_3_ ratios lowered the viscosity of slag at the experimental temperature, and the decrement in viscosity at high temperatures was not obvious. This is consistent with the previous studies [10,18,19,20], where MgO is classified as a basic oxide similar to CaO in the silicate melts, and it acts as a network modifier. Furthermore, MgO could provide free oxygen ions to cut down the Si–O bonds in the network structure, resulting in the decrease in the DOP of the slag structure, expressed as Equations (10) and (11) [4,21]. These transition processes could improve the fluidity of molten slag.

[Si_3_O_9_]^6−^ (ring) + O^2−^ = [Si_3_O_10_]^8−^ (chain) (10)

[Si_3_O_10_]^8−^ + O^2−^ = [Si_2_O_7_]^6−^ + [SiO_4_]^4−^ (monomer) (11)

**Figure 7:**
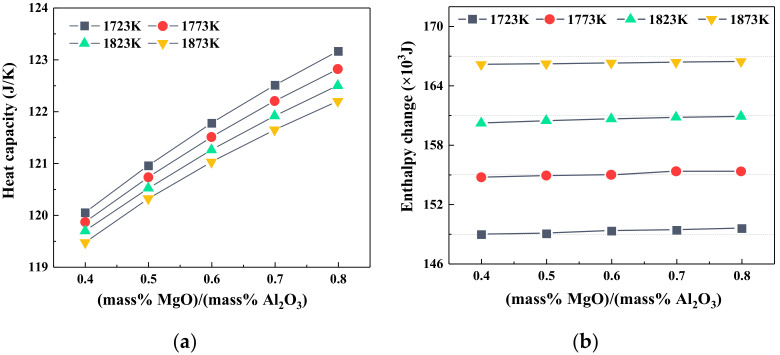
The heat capacity and enthalpy change of slag with different MgO/Al_2_O_3_ ratios; (**a**) heat capacity; (**b**) enthalpy change.

The effects of the MgO/Al_2_O_3_ ratio on the heat capacity and enthalpy change of slag at various temperatures are depicted in Figure 7. It shows that the increment in MgO/Al_2_O_3_ led to a larger heat capacity of slag at the constant temperature, and the increment in heat capacity was much larger than that caused by the addition of TiO_2_. The enthalpy changes of slag also increased with increasing MgO/Al_2_O_3_ ratios and temperature. However, the absolute values of the enthalpy change at the experimental temperature only increased slightly with increasing MgO/Al_2_O_3_ ratios, which was basically consistent with the previous study. It was reported in the work [19] that a proper increase in the MgO/Al_2_O_3_ ratio was beneficial to reduce the fuel ratio or coke ratio of the blast furnace to some extent.

**Figure 8:**
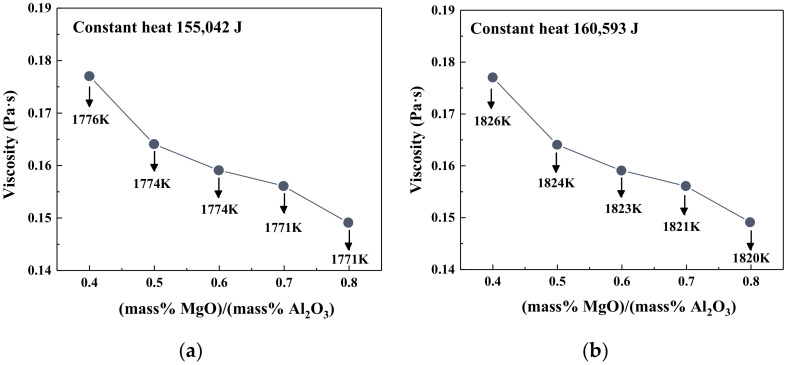
The viscosity of slag with different MgO/Al_2_O_3_ ratios under constant heat quantity: (**a**) 155,042 J; (**b**) 160,593 J.

Figure 8 shows the viscosity and temperature of slag as a function of MgO/Al_2_O_3_ ratios under fixed heat input conditions. The calculated heat input quantities were 155,042 J (1773 K) and 160,593 J (1823 K), respectively. It could be seen that the temperature in slag decreased with increasing MgO/Al_2_O_3_ ratios, and the average decrement of slag temperature was 5 K. However, it is interesting to note that although the temperature of slag decreased, the viscosity had not increased but decreased. This showed that the influence of the slag composition on the viscosity was more pronounced than the slight decrease in temperature under a fixed heat supply. Therefore, the energy consumption of the BF could be reduced by appropriately increasing the MgO/Al_2_O_3_ ratio of slag.

### 3.3. Effect of TiO_2_ and MgO/Al_2_O_3_ Ratio on the Activation Energy for Viscous Flow

Generally, the activation energy represents the energy barrier to overcome when the liquid melt flows, and the variation in activation energy reflects the changes to slag structures. This can be calculated from the Arrhenius-type equation [22,23], as expressed by Equation (12).

(12) $$\eta \;=\;\mathrm{AT}\;\exp\;(\frac{E_{a}}{\;\mathrm{RT}\;})$$

Equation (12) can be written as Equation (13) after taking the logarithm.

(13) $$\ln(\frac{\eta }{T})\;=\;\mathrm{lnA}\;+\;\frac{E_{a}}{\mathrm{RT}}$$

where η is the viscosity of the melt, (Pa·s); A is the pre-exponent factor, and E_a_ is the apparent activation energy of the slags, (J/mol); R is the gas constant, (8.314 J/mol·K); T is the absolute temperature, (K). 

**Figure 9:**
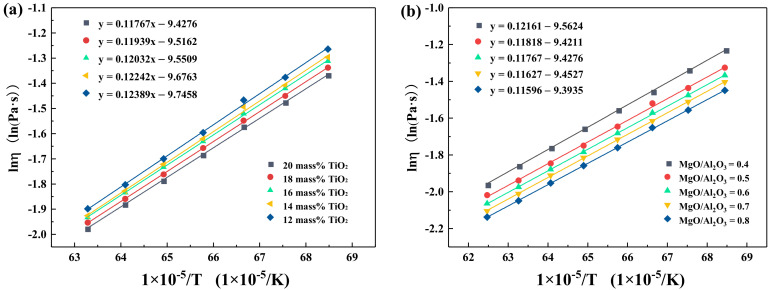
Temperature dependence of the viscosity of the slags (**a**) with varying TiO_2_ content; (**b**) with varying MgO/Al_2_O_3_ ratio.

From Equation (4), ln( $\frac{\eta }{T}$ ) has a linear relationship with $\frac{1}{\mathrm{RT}}$, which is shown in Figure 9. The slope of the fitting line (R^2^ > 0.95) is the apparent activation energy E_a_. The calculated results of apparent activation energy are listed in Table 2. As expected, the apparent activation energy decreased from 123.89 kJ/mol to 117.67 kJ/mol with the TiO_2_ content from 12 to 20 mass%, and it also decreased from 121.61 kJ/mol to 115.96 kJ/mol with the increment of the MgO/Al_2_O_3_ ratio from 0.4 to 0.8. This indicated that the frictional resistance of melts and the fraction of complex structural units decreased. The results were consistent with the variation of viscosity.

### 3.4. Effect of TiO_2_ on the Structure Using FTIR Spectroscopy

**Figure 10:**
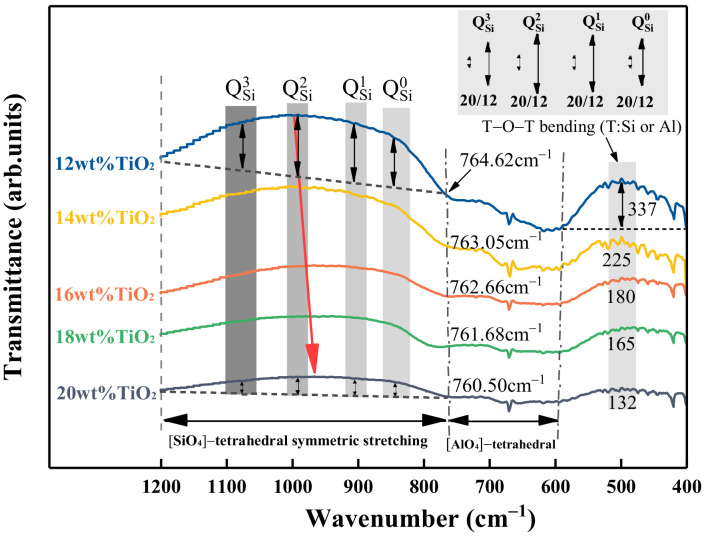
Effect of TiO_2_ on the results of FTIR spectra of as-quenched samples.

The FTIR spectra of the quenched slag were measured to obtain information about the slag structure. The original FTIR spectra for the amorphous samples with different TiO_2_ content at fixed basicity of 1.10 are presented in Figure 10. According to the previous studies [24,25,26], the vibration bands of silicate are assigned to the range of 1200 to 400 cm^−1^. The spectra were divided into three wavenumber bands: 1200–760 cm^−1^, 760–600 cm^−1^, and 600–400 cm^−1^. For [SiO_4_]-tetrahedral bands, it was divided into four typical silicate structural units Q^n^ (n = 0, 1, 2, 3, which represents the number of bridge oxygen atoms per silicate tetrahedral structure) and represented the DOP of the melted structure. The assignment of each band is shown in Table 3. 

From Figure 10 and Table 3, as the TiO_2_ content increased, the trough of [SiO_4_]-tetrahedral symmetric stretching bands located at 800–1200 cm^−1^ became less pronounced, and the depth of the transmission trough got shallower. Furthermore, the band center of the [SiO_4_]-tetrahedral shifted from 996.37 cm^−1^ to 965.32 cm^−1^ as the TiO_2_ content increased. This indicated that the intricate three-dimensional silicate network structures were depolymerized and formed simpler structural units. In addition, the asymmetric stretching vibration bands for the [AlO_4_]-tetrahedron dampened with increasing TiO_2_ as well, and the wavenumber of [AlO_4_]-tetrahedron bands gradually decreased from 764.62 cm^−1^ to 760.50 cm^−1^. It suggested that TiO_2_ also played a role in depolymerizing the aluminate structure. According to Xu et al. [31], Ti^4+^ might incorporate into the slag structure and substitute some Al^3+^ in [AlO_4_]^5−^ to form [TiO_4_]^4−^, but cations (e.g., Ca^2+^) are needed to keep the equilibrium of electric charge. When Ti^4+^ substituted Al^3+^, [TiO_4_]^4−^ generated and [AlO_4_]^5−^ disappeared, which meant less cations consumption. Thus, the oxide (e.g., CaO) acted as network modifier, accordingly increased, and viscosity decreased. The depth of the T–O–T (T donates Si, Al, or Ti) bending band significantly decreased with increasing TiO_2_ content. This further illustrated a decrease in the linkage between [SiO_4_]-tetrahedron and [SiO_4_]-tetrahedron or [SiO_4_]-tetrahedron and [AlO_4_]-tetrahedron, and the complex structural units were depolymerized. This result is because [TiO_4_]-tetrahedral units entered melts and incorporated silicate structures, and Si–O–Ti units were formed according to Equation (14). Since the electrostatic potential of Ti^4+^ (1.85 I) is lower than that of Si^4+^ (2.51 I), the bond strength of Ti–O is weaker than Si–O, and the stability of slag weakens [32,33]. In addition, according to the stable energy concept proposed by Duan et al. [34], the stable energies of [TiO_4_] and [SiO_4_] were calculated to be approximately 7.282 × 10^−5^ and 8.426 × 10^−5^ kJ·mol^−1^, respectively. It suggested that [TiO_4_] was more easily destroyed than [SiO_4_], which meant that the slag needed to absorb less heat energy, that is, the heat capacity of slag decreased. Therefore, the heat capacity and viscosity of slag decreased. The reduction in viscosity indicated that TiO_2_ played the role of a network modifier to weaken the complexity of the network structure. The comprehensive effects of TiO_2_ may eventually cause the decrease in slag viscosity.

Si−O−Si + 2[TiO_4_] → 2Si−O−Ti (14)

## 4. Conclusions

The effects of TiO_2_ content (12 mass% to 20 mass%) and MgO/Al_2_O_3_ (0.4 to 0.8) ratios on the viscosity, heat capacity, and enthalpy change of Ti-bearing BF slag at a constant heat supply were investigated, and the structure of slag with varying TiO_2_ contents was studied by using FTIR spectroscopy. The following conclusions could be drawn.

The viscosity of slag decreased as the TiO_2_ content and MgO/Al_2_O_3_ ratio increased at a constant temperature, and the heat capacity and enthalpy change both decreased with increasing TiO_2_ content. With the increase in the MgO/Al_2_O_3_ ratio, the heat capacity of slag increased, and the absolute values of the enthalpy change only increased slightly at the experimental temperature.

1. At the fixed heat quantity supply, the increase in TiO_2_ content led to an increase in slag temperature but a decrease in the viscosity. As the MgO/Al_2_O_3_ ratio increased, the temperature of slag had the opposite variation trend, but the viscosity of slag still decreased.
2. The apparent activation energy decreased from 123.89 kJ/mol to 117.67 kJ/mol with the increment of TiO_2_ content, and it also decreased from 121.61 kJ/mol to 115.96 kJ/mol with higher MgO/Al_2_O_3_ ratios.
3. The vibration bands of [SiO_4_]-tetrahedron, [AlO_4_]-tetrahedron, and T–O–T became less pronounced. TiO_2_ played a role in weakening the stability of the molten slag structure and depolymerizing the complex network structural units into simpler structures, which eventually decreased the viscosity.

## Figures and Tables

**Table 1:** Experimental compositions of slags (mass%).

No.	CaO	SiO_2_	Al_2_O_3_	MgO	TiO_2_	MgO/Al_2_O_3_	Liquid Temperature (K)
1	30.03	27.30	14.17	8.50	20	0.6	1432
2	31.08	28.25	14.17	8.50	18	–	1430
3	32.13	29.20	14.17	8.50	16	–	1427
4	33.17	30.16	14.17	8.50	14	–	1421
5	34.22	31.11	14.17	8.50	12	–	1413
6	30.03	27.30	16.19	6.48	20	0.4	1440
7	30.03	27.30	15.11	7.56	20	0.5	1436
8	30.03	27.30	13.34	9.33	20	0.7	1428
9	30.03	27.30	12.59	10.08	20	0.8	1424

**Table 2:** The calculated results of apparent activation energy.

TiO_2_ (mass%)	E_a_ (kJ/mol)	MgO/Al_2_O_3_ (mass%/mass%)	E_a_ (kJ/mol)
20	117.67	0.4	121.61
18	119.39	0.5	118.18
16	120.32	0.6	117.67
14	122.42	0.7	116.27
12	123.89	0.8	115.96

**Table 3:** Assignments of FTIR spectra bands associated with structural units.

Wavenumber (cm^−1^)	Assignments	References
400–600	T-O-T bond bending vibrations (T denotes Si, Al, or Ti elements, etc.)	[17,18,27,28]
600–760	The symmetric stretching vibration of [AlO_4_]-tetrahedron	[14,18,29,30]
~850	Q^0^ ([SiO_4_]^4^^−^ monomers)	[14,16,29,30]
~900	Q^1^ ([Si_2_O_7_]^6^^−^ dimers)	[14,16,29,30]
~950–1000	Q^2^ ([Si_3_O_10_]^8^^−^ chains)	[14,16,29,30]
~1050	Q^3^ ([Si_3_O_9_]^6^^−^ rings)	[14,16,29,30]

## Data Availability

The data presented in this study are available on request from the corresponding author.
